# Milk Polar Lipids: Underappreciated Lipids with Emerging Health Benefits

**DOI:** 10.3390/nu12041001

**Published:** 2020-04-04

**Authors:** Liya Anto, Sarah Wen Warykas, Moises Torres-Gonzalez, Christopher N. Blesso

**Affiliations:** 1 Department of Nutritional Sciences, University of Connecticut, Storrs, CT 06269, USA; liya.anto@uconn.edu (L.A.); sarah.warykas@uconn.edu (S.W.W.); 2 National Dairy Council, Rosemont, IL 60018-5616, USA; moises.torres-gonzalez@dairy.org

**Keywords:** polar lipids, dairy, sphingomyelin, heart disease, gut health, cancer, inflammation

## Abstract

Milk fat is encased in a polar lipid-containing tri-layer milk fat globule membrane (MFGM), composed of phospholipids (PLs) and sphingolipids (SLs). Milk PLs and SLs comprise about 1% of total milk lipids. The surfactant properties of PLs are important for dairy products; however, dairy products vary considerably in their polar lipid to total lipid content due to the existence of dairy foods with different fat content. Recent basic science and clinical research examining food sources and health effects of milk polar lipids suggest they may beneficially influence dysfunctional lipid metabolism, gut dysbiosis, inflammation, cardiovascular disease, gut health, and neurodevelopment. However, more research is warranted in clinical studies to confirm these effects in humans. Overall, there are a number of potential effects of consuming milk polar lipids, and they should be considered as food matrix factors that may directly confer health benefits and/or impact effects of other dietary lipids, with implications for full-fat vs. reduced-fat dairy.

## 1. Introduction

Polar lipids are essential components of all biological membranes and found in the human diet as phospholipids (PLs) and sphingolipids (SLs). Consumption of dietary polar lipids is relatively common in the Western dietary pattern and estimated to be in the range of 2–8 g/day for PLs (~1–10% of daily fat intake) [1] and 50–400 mg/day for SLs [2,3]. In milk, polar lipids are primarily located within the milk fat globule membrane (MFGM), which is a trilayered biological membrane that surrounds the fat globule. MFGM are typically derived from cell membranes of lactating cells and the endoplasmic reticulum membranes [4]. Animal cell membranes, in general, have PLs as the major structural lipids and SLs, such as sphingomyelin (SM), are typically components of lipid rafts in association with cholesterol [5]. Of the total milk lipids, polar lipids account for approximately 1% of total lipids in milk. SM content encompasses approximately 25% of total milk polar lipids, and SM is found at ~3:1 ratio to cholesterol by mass [6]. While health effects of PLs from eggs [7] and SLs [8] have been reviewed previously, the health effects of consuming milk polar lipids have not been reviewed extensively. Since the SLs found within MFGM are known to impact various aspects of lipid metabolism [9,10], gut microbiota [10], and inflammation [9,11], milk polar lipids may be considered as food matrix factors that may confer health benefits and/or impact effects of other dietary lipids, with implications for full-fat vs. lower-fat dairy varieties. This review summarizes the recent basic science and clinical research examining food sources and health effects of milk polar lipids, as well as to identify gaps in the scientific literature related to milk polar lipids research.

## 2. Polar Lipids

### 2.1. Classes of Polar Lipids

**Figure 1:**
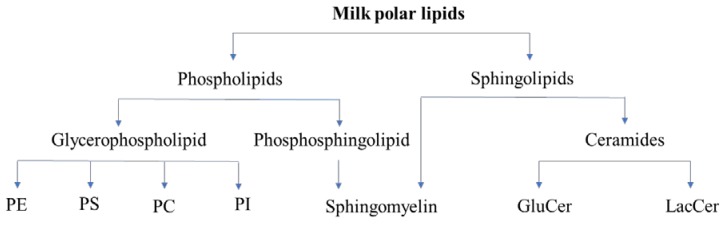
Classification of milk polar lipids. Abbreviations: GluCer, glucosylceramide; LacCer, lactosylceramide; PC, phosphatidylcholine; PE, phospatidylethanolamine; PI, phosphatidylinositol; and PS, phosphatidylserine.

Phospholipids and sphingolipids are the two major classes of polar lipids in milk. PLs in milk includes SM (considered both a PL and a SL) and glycerophospholipids. The glycerophospholipids consist of phosphatidylethanolamine (PE), phosphatidylserine (PS), phosphatidylcholine (PC), and phosphatidylinositol (PI). Sphingomyelin is a phosphosphingolipid and is the major SL found in milk. Other SLs in milk include glucosylceramide (GluCer) and lactosylceramide (LacCer). Classification of milk polar lipids is given in Figure 1.

Glycerophospholipids are amphipathic molecules comprised of a glycerol backbone, two ester-linked fatty acids (FAs) which form the hydrophobic tail (at sn-1 and sn-2 positions of glycerol), and a polar head group linked by a phosphate residue (at sn-3 position). The polar head group can be ethanolamine, serine, choline, or inositol, which makes it PE, PS, PC, or PI, respectively [12]. Fatty acids seen in glycerophospholipids are usually unsaturated and long chain. However, PC is reported to have more saturated FAs when compared with other PLs seen in milk [6]. Esterification of very-long-chain fatty acids in glycerophospholipids is also reported, although to a lesser extent than long-chain FAs [13].

Sphingolipids are polar molecules that differ from glycerophospholipids in that instead of glycerol, they contain a long-chain amino alcohol, known as a sphingoid base, as their backbone [14]. The predominant mammalian sphingoid base is the 18-carbon sphingosine. Ceramide is formed when the amino group of sphingosine is linked to a FA via an amide bond. Sphingomyelin (or ceramide phosphocholine) is an amphipathic sphingolipid in which a phosphorylcholine head group is linked to ceramide [15]. In glucosylceramide (GluCer), the 1-position of ceramide is linked to a glucose residue, whereas in lactosylceramide (LacCer), it is linked to lactose [8,16]. 

Previous literature has cited the presence of other minor PLs in milk, including lysophosphatidylethanolamine (LPE) and lysophosphatidylcholine (LPC) [17,18,19]. However, the origin of these lipids in milk is unclear, as they are the hydrolytic products of PE and PC and can be formed by hydrolysis during milk handling [12]. Another minor class of PLs seen in milk are the plasmalogens, which are structurally characterized as a glycerophospholipid having a vinyl-ether linkage with a fatty alcohol at the sn-1 position of the glycerol backbone [20,21].

### 2.2. Biological Functions

Phospholipids are present in all biological membranes, where they display amphipathic properties. The amphipathic nature of PLs is characterized by two hydrophobic tails and a hydrophilic, polar head group. This feature helps them to aggregate spontaneously in the aqueous phase by exposing their hydrophilic head and hiding their hydrophobic tails away from the aqueous phase, thereby attaining an energetically favorable conformation [22]. Phospholipids aggregate in two different ways; they can either bury their hydrophobic tails in the interior forming a spherical structure, as seen in micelles, or they can sandwich their tails between the hydrophilic head groups forming sheets or bilayers, as seen in cell membranes [23]. 

Almost 50% of the total mass of the animal cell membrane is made up of lipids, with PLs being the most abundant. The major PLs in cell membranes include PC, PE, PS, and SM. They serve a wide variety of structural and biological functions in the cell membrane [23]. Phospholipid bilayers in cell membranes help in maintaining a semi-permeable barrier between the cells and the organelles [24,25]. Due to their amphipathic nature and cylindrical structure, the PLs in cell membrane can also demonstrate a self-healing property. A tear in the membrane can produce an energetically unfavorable form, which compels the PLs to rearrange spontaneously to heal/close the tear [23]. This specific property of PLs is fundamental to the existence of a cell. Within the lipid bilayers, individual lipids can move freely, which provides fluidity to the membrane [26]. The presence of PS in the cytosolic surface of the bilayer is important for the enzyme activity of cytosolic protein kinase C. Phospholipids are important intracellular mediators. Extracellular signals can activate various membrane phospholipases that can cleave PLs and the fragments produced can act as intracellular mediators [24,27,28]. Thus, PLs are integral parts of cellular membranes and are also critically involved in vital functions of the cell. 

During lipid digestion in the small intestine, mixed micelles are formed, which also contain PLs on their surface. As previously mentioned, the spherical structure of the micelle is due to the amphipathic property of PLs. Phospholipids are arranged in micelle in such a way that the hydrophilic head group face to the exterior, while the hydrophobic tails are arranged on the inside. This particular arrangement of PLs, along with bile salts, helps in the packing of highly hydrophobic triglyceride (TG) and cholesteryl esters in the core of mixed micelle, so that they can be transported through the aqueous environment in the intestine where they can be digested and absorbed. Likewise, the emulsifying function of PL monolayers in lipoproteins derived from the intestine and liver is similar to what is observed in micelles. 

### 2.3. Milk Polar Lipids: Classes and Quantity

Not all dairy products are created equal when it comes to PL and SL contents. There is a considerable amount of variability when comparing polar lipids to total lipid content in dairy products. For example, some fat-rich products like butter and cream have relatively low levels compared to other products. For the content of SLs, which is mostly SM, dairy products vary from trace levels of SM in anhydrous milk fat to higher levels in buttermilk (~12–21.5 g/100 g of PL) and butter serum (also called beta serum) (23.8–28.92 g/100 g of PL) [29]. The major PL and SM content of raw milk and other dairy products is summarized in Table 1. Whole milk contains about twice the PLs of skim milk, while some dairy co-products can become relatively enriched in PLs, including buttermilk and butter serum [29]. Phospholipid content in whole milk can vary between 0.7 to 2.3 g/100 g of fat [30,31,32,33,34]. This variation in the level of reported PL content in milk can be attributed to the different analytical techniques used, as well as the diet [35], season of the year [36], age, and stage of lactation of the animal [37,38]. The most abundant PL in milk is reported to be PE, followed by PC and SM [20,29,31,33,34,37,39,40,41]. Fat-rich dairy products like butter and cream are found to be less enriched in PL content as a proportion of total fat, when compared with their aqueous co-products. Cream is rich in fat (~32%) and protein (~2%) [31] and has 0.3–5.65 g of PL/100 g of fat. Most authors reported PE being the most abundant PL found in cream, followed by PC and SM [30,32,42,43]. Butter (~78% fat) [32] has a PL content ranging from 0.195–5.31 g/100 g fat. The order of abundance of PL in butter is the same as cream, with PE being the richest followed by PC and SM [30,31,32,44]. Buttermilk (4.485–35.32 g/100 g of fat) and butter serum (46.69–48.39 g/100 g of fat), which are the co-products of butter and anhydrous milk fat production, respectively, are rich sources of polar lipids, yet are low in fat [32]. Cheese whey is also a good source of milk PL (5.3–23.66 g/100 g fat). 

It has been reported that various dairy production methods (homogenization, heating, etc.) can disrupt the MFGM [30]. They can also affect the MFGM protein content in the final product and will enhance the milk PL content in the serum phase [51,52,53]. In addition, several reports have shown that milk products which are rich in PL are also rich in MFGM proteins [6,30,54,55,56]. As a consequence, it is suggested that, due to the close association of milk PL with MFGM proteins, these fractions may be migrating together during dairy processing [6]. Churning, centrifuging, homogenization, and spray-drying are some of the dairy processing techniques which are known to affect the composition of milk PLs and SLs [30]. This could be one probable reason behind this unexpected inverse relationship between fat and milk PL content in dairy ingredients and co-products. Due to the high PL content on a dry matter and fat base, buttermilk, butter serum, and cheese whey are the most suitable sources of milk PL purification. However, these ratios are much higher for butter serum when compared with buttermilk and cheese whey, which makes it the most attractive source for milk PL purification [49]. However, researchers have also successfully used buttermilk [57,58] and cheese whey [56] for the extraction of milk PLs. 

## 3. Health Effects of Milk Polar Lipids

### 3.1. Effects on Intestinal Lipid Absorption 

Dietary PLs can inhibit intestinal lipid absorption when added to the diet in significant amounts by interfering with lipid mobilization from mixed micelles (as reviewed by Cohn J et al. [1]). Dietary SM, in particular, is known to dose-dependently reduce the intestinal absorption of cholesterol, TG, and fatty acids in rodents [10]. Products of SM digestion, such as ceramides and sphingosine, also inhibit cholesterol and fatty acid absorption [59,60,61]. Compared to SM derived from egg yolk, SM from milk has been shown to have stronger effects on inhibiting the intestinal absorption of fat and cholesterol in rats, potentially due to stronger hydrophobic interactions between milk SM and other lipids [62]. Evidence of dietary SM and its hydrolytic products inhibiting intestinal lipid absorption through cell-dependent and cell-independent mechanisms is provided in previous preclinical studies [63]. Enrichment of the dietary PL pool by milk SM has shown to decrease intestinal cholesterol absorption by decreasing the active concentration of cholesterol monomers in mixed micelles [64]. In vitro studies suggest that SM in mixed micelles can reduce TG hydrolysis by inhibiting human pancreatic lipase-colipase activity [65,66]. Long-chain bases of SLs and long-chain fatty acids also have been reported to compete with each other for cellular uptake, since they both utilize acyl-CoA synthetases [61].

Other milk polar lipids such as PC, PE, and gangliosides are also known to reduce the intestinal absorption of dietary lipids. Phosphatidylcholine, which when present in the bile can facilitate intestinal absorption of dietary lipids, can inhibit lipid absorption when present in large amounts in the diet. The presence of PC in taurocholate containing mixed micelles reduced the uptake of cholesterol by Caco-2 cells [67]. Incubation of Caco-2 cells with micelles containing 200 µM of PC reduced cholesterol absorption, accompanied by reduced cellular esterification and secretion of cholesteryl esters. In contrast, the presence of lysophosphatidylcholine showed only a minor effect. Additionally, previous research also revealed the capacity of intact PC in mixed micelles to inhibit the absorption of cholesterol and FAs using in vivo (isolated jejunal segment technique) and in vitro (everted sac experimental model from rat jejuna) studies [68]. Similar effects of PC are also reported by Rampone and Long (1977) using the same in vitro model [69]. In human trials, the administration of PC via intraduodenal infusion attenuated cholesterol absorption when compared to the placebo group, which received the same amount of safflower oil with similar FA composition [70]. Later studies showed that phospholipase A2 hydrolysis of surface PL in lipid emulsions prior to pancreatic lipase/co-lipase-mediated TG hydrolysis is necessary for the cellular uptake of cholesterol and FAs. The cellular uptake of cholesterol from lipid emulsions with high PL/TG molar ratio (>0.3) was significantly lower [71]. However, the exact mechanism by which high PC concentration reduces intestinal cholesterol absorption remains unclear. Although, a plausible mechanism can be attributed to an increased solubility of cholesterol in the micelle and, thus, shifting the partition coefficient away from the cell membrane [72]. Additionally, a higher concentration of PC in lipid emulsions may also lead to a decreased availability of cholesterol for absorption, due to the increased packing density of the micellar surface [71].

Phosphatidylethanolamine is shown to have a hypocholesterolemic effect in animal studies [73,74]. Supplementation with 2% PE for two weeks in rats fed with a 1% cholesterol-containing diet reduced serum cholesteryl ester compared to control animals, with the cholesteryl ester concentration being inversely related to the level of hepatic PE [74]. A similar effect of PE in rats was also reported by Imaizumi and co-workers [73]. It is also known that mono- and di-unsaturated PE can exhibit a similar affinity to cholesterol as that of SM and can influence cholesterol absorption [75,76]. Therefore, PE found in milk may also have the capability to reduce intestinal lipid absorption, due to its affinity for cholesterol; however, further studies are needed to confirm this.

### 3.2. Anti-Inflammatory Effects

Dietary SM and its hydrolytic products (i.e., sphingosine and ceramide) have shown promising anti-inflammatory action in preclinical studies. Mazzei et al. [77] showed that sphingosine can activate a peroxisome proliferator-activated receptor-gamma (PPARγ) reporter in macrophages. PPARγ is a nuclear receptor that represses the transcriptional activation of inflammatory response genes in mouse macrophages [78]. Similarly, consumption of milk SM effectively decreased disease activity and colonic inflammatory lesions in mice with chemically induced colitis, partly through PPARγ [77]. Norris et al. [11] found that milk SM was not cytotoxic to RAW264.7 macrophages at physiological dosages tested but strongly decreased LPS-stimulated pro-inflammatory gene expression. The major bioactive component in these experiments was sphingosine, as only sphingosine and sphingosine-containing SLs recapitulated the anti-inflammatory effects of milk SM. Consistent with this finding, sphingosine reduced TNF-α production from macrophages stimulated with LPS [79]. The choline moiety of milk PLs may also contribute some protection against macrophage inflammation. Choline dose-dependently reduced TNF-α release from macrophages stimulated with endotoxin by inhibiting NF-κB activation [80]. In mice, intraperitoneal injection of choline (50 mg/kg) prior to the endotoxin treatment significantly improved the survival rate and decreased plasma TNF-α level [80]. This anti-inflammatory effect of choline was found to be nicotinic acetylcholine receptor subunit α7 (α7nAChR)-dependent, which is an essential component of the cholinergic anti-inflammatory pathway [80]. Collectively, these data demonstrate that milk SM has potential to be anti-inflammatory in macrophages.

Milk polar lipids and their metabolic products also appear effective against endotoxemia. Endotoxemia is a persistent sub-clinical, low-grade inflammation due to circulating endotoxins, primarily LPS, which may be absorbed into circulation due to defects in the gut barrier. Milard et al. [81] observed gut barrier effects when Caco-2/TC7 cells were treated with milk SM incorporated into mixed micelles. They observed significant inductions in the gene expression of tight junction proteins, which was related to a specific induction of interleukin-8 (IL-8) by milk SM. While IL-8 has been reported to have pro-inflammatory effects in some conditions, this study showed recombinant IL-8 specifically increased tight junction protein expression. More research is warranted to investigate the role of milk SM on IL-8 and gut barrier function. While these effects were observed with milk SM, some studies have been conducted with MFGM as a source of polar lipids. Snow et al. [82] studied the effect of MFGM on gut-barrier and systemic inflammation in LPS-challenged mice by feeding them with 10% MFGM containing diet (6 g of milk polar lipids/kg of the diet). The MFGM supplemented diet significantly attenuated LPS inducted systemic inflammation, partly by improving gut barrier integrity. Moreover, a four-week parallel intervention study in healthy adults showed that consumption of 10 g of a commercially-available MFGM-rich milk protein concentrate (Lacprodan PL-20, containing 16% PL by weight) twice daily can provide in vivo resistance to food-borne infections [83]. Norris et al. [10] was the first to report that circulating LPS activity was reduced in high-fat diet (HFD)-fed C57BL/6J mice supplemented with milk SM (0.25% *w*/*w*) for 4 weeks. Additionally, supplementation of milk SM (0.1% *w*/*w*) to an obesogenic diet dramatically reduced serum inflammatory cytokines/chemokines and mRNA expression of inflammation markers in adipose tissue in C57BL/6J mice [9,11]. Accordingly, Li et al. [84] reported that supplementation of MFGM (200 mg/kg body weight) for eight weeks attenuated HFD-induced intestinal inflammation, improved gut barrier tight junction protein expression, and reduced LPS activity and inflammation biomarkers in the circulation of C57BL/6 mice.

### 3.3. Modulation of Gut Microbiota

An emerging area of importance is the potential modulation of gut microbiome by dietary polar lipids [63]. Humans harbor trillions of bacteria in their gastrointestinal (GI) tract as part of the natural gut microbiota [85]. The residency of these microbes in the GI tract is closely linked with human physiology and plays a vital role in the function of the gut. The three most abundant phyla in human gut microbiome are Bacteroidetes, Firmicutes, and Actinobacteria [86]. Both Firmicutes and Bacteroidetes make up about 90% of phyla in the human gut microbiota [87]. The significance of the Firmicutes/Bacteroidetes ratio to human health has yet to be fully elucidated, yet it appears that lean individuals have a greater proportion of gut microbiota as Bacteroidetes compared to obese individuals, whereas the opposite is seen for Firmicutes [88]. Thus, the Firmicutes/Bacteroidetes ratio is often used as a marker of gut dysbiosis related to obesity and HFD consumption. Bifidobacterium belongs to the phylum Actinobacteria, and it is the most abundant bacteria seen in the gut of breastfed infants [89,90]. Lower levels of gut bifidobacteria are commonly associated with many disease conditions, including hepatitis B [91], cystic fibrosis [92], Type 1 and Type 2 diabetes [91,93], and obesity [94,95].

The SL fraction of milk polar lipids are reported to possess antibacterial effects. Sprong et al. [96] were the first to report the antibacterial effect of milk SLs. In their study, they showed the antibacterial effects of galactosylsphingosine and lysoSLs against Gram-positive and Gram-negative bacteria. In addition, Fischer et al. [97] showed that sphingoid bases (e.g., sphingosine, phytosphingosine, and dihydrosphingosine) also have broad-spectrum antimicrobial activity, which was supported by the induction of ultra-structural damage subsequent to pathogen uptake [98]. Nejrup et al. [99] conducted a 24 h in vitro fermentation study in the fecal sample of healthy infants to determine the effect of digestive products of milk lipids on modulating gut bacteria. They found that long-chain non-esterified fatty acids (LC-NEFA) with 10% sphingosine can increase bifidobacteria relative abundance in fecal content, whereas the LC-NEFA alone did not have an influence on bifidobacteria populations [99]. These in vitro findings suggest that milk SLs and their metabolic products may potentially exert changes in the gut microbiota when consumed regularly. Milk SM is digested and absorbed in the middle and distal part of the small intestine in rats and, presumably, in humans [100]. However, a large fraction of dietary SM and its digestive products reach the colon [101], where they may exert their bactericidal and gut-modulating effects. The effects of milk PLs on gut microbiota composition in both pre-clinical and clinical studies are summarized in Table 2.

Norris et al. [10] first reported the gut microbiota modulating effects of purified milk SM. Supplementation of a HFD (45% kcal as fat) with 0.25% (*w*/*w*) milk SM for four weeks increased the relative abundances of fecal Bifidobacterium, Firmicutes, and Actinobacteria, as well as reduced Bacteroidetes in C57BL/6J mice [10]. Supplementation of 0.25% (*w*/*w*) milk SM to a semi-purified low-fat diet also increased fecal Bifidobacterium in C57BL/6J mice [63]. However, a longer supplementation (10 weeks) of a lower dose of SM (0.1% milk SM) in mice fed an obesogenic diet (60% kcal as fat) had weaker effects on gut microbiota, with little change except for an increase in Acetatifactor relative abundance [11]. 

With SM being the most bioactive component in milk PLs, effects of the total milk polar lipids fraction on gut microbiota have shown similar results to the animal studies using purified SM. We have recently observed that feeding 2% milk PLs (containing 0.4% milk SM) to HFD-fed LDL-receptor knockout mice resulted in a similar increase in fecal Bifidobacterium relative abundance, as previously observed by Norris et al. [102]. Interestingly, there was also an increase in the Bacteroidetes phylum, which significantly reduced the Firmicutes to Bacteroidetes ratio in the 2% milk PL-supplemented group [102]. Milard et al. [103] found that supplementing an HFD with 1.6% milk PLs (0.38% SM) induced a reduction in fecal Lactobacillus in C57BL/6 mice, while 1.1% milk PLs (0.25% SM) induced an increase in Bifidobacterium compared with the HFD-fed controls. Akkermansia muciniphila, which is classified under the Verrucomicrobia phylum, was also significantly higher in milk PL-fed mice. Akkermansia muciniphila is noted for its positive metabolic effects, which includes improving insulin sensitivity and protecting against metabolic endotoxemia-induced inflammation [104,105,106]. In addition, there was a significant positive correlation between Bifidobacterium animalis and Akkermansia muciniphila for the milk PL-supplemented mice [103]. However, a study conducted by Reis et al. [107] did not find any effect on the gut microbiota composition by supplementing total polar lipids, PLs, or SLs in HFD-fed C57BL/6J mice. This particular experiment by Reis et al. [107] differed from other experimental designs, as mice were first fed with HFD for five weeks and then later supplemented milk polar lipids along with the HFD for an additional five weeks. As the HFD consumption likely resulted in altered gut microbiota after five weeks, the delay in polar lipid treatment may have contributed to the insignificant effects in this case. Modulation of the gut microbiota by MFGM, i.e., the milk PL-rich fraction of milk, has also been tested in rodents. The pup-in-a-cup model was used in five-day-old rats to examine the feeding of formula with fat from vegetable sources only, formula with supplemented MFGM, or mother’s milk on intestinal development and the gut microbiota [108]. After 10 days, the pups fed a formula supplemented with MFGM had a more similar intestinal development and gut microbiota to those fed mother’s milk when compared to those fed formula only. In another study, Li et al. [84] reported that supplementation of MFGM (200 mg/kg body weight) to C57BL/6 mice for eight weeks attenuated gut dysbiosis that occurs with HFD, including increasing the Bacteroidetes/Firmicutes ratio. Recently, modulation of the gut microbiome by ethanolamine, which is the base constituent of PE, was tested by supplementing 0, 250, 500, and 1000 µM ethanolamine to the drinking water of rats [109]. Ethanolamine supplementation at 500 and 1000 µM significantly increased Bacteroidetes and decreased Proteobacteria, Elusimicrobia, and Tenericutes. In addition, a reduction in Spirochetes was also noticed in the mice provided with 500 µM ethanolamine [109]. Overall, most pre-clinical studies in mice have noted some impact of milk polar lipids on gut microbiota composition; however, there are differences in microbiota profiles across studies. The observed differences between studies may be related to varying dosages, forms of milk polar lipids, base diet composition, or the use of prevention or treatment models.

While the pre-clinical findings reporting modulation of the gut microbiome by milk polar lipids are promising, only one human clinical trial has been conducted investigating this area. In a recent study by Vors et al. [110], post-menopausal women who supplemented their diets for four weeks with either 3 g or 5 g of milk polar lipids daily through butter serum showed no significant changes in major phylogenetic groups or bacterial species of gut microbiota when compared with the control group fed only butter oil. This may be attributed to the much lower dosage used in this study when compared to animal studies. A simple allometric approach considering the body surface area can be used to convert mice dose in mg/kg to human equivalent dose (mg/kg) by multiplying by 0.081 [111]. For example, the animal dose of milk PLs in Millar et al. [102] shown to modulate gut microbiota was ~1.25–2.5 g PLs/kg of body weight, which is equivalent to ~0.1–0.2 mg/kg of bodyweight in humans. This would equate to a dose of 7–14 g of milk PLs in a 70 kg human. However, Vors et al. [110] reported there was a significantly greater amount of fecal coprostanol, as well as a higher coprostanol/cholesterol ratio in those supplemented with milk polar lipids compared to control. While not examined further, these effects suggest there were changes in gut microbiome metabolism specific to increased coprostanol conversion in the gut with milk polar lipid supplementation. Some gut microbes are known to have the ability to convert cholesterol to coprostanol [112,113]. Research has shown an inverse relationship between blood cholesterol concentrations and the coprostanol/cholesterol ratio in human feces, suggesting the ability of coprostanol to modulate cholesteremia [114]. Thus, metagenomic effects of milk polar lipids and their influence on coprostanoligenic bacteria warrant more investigation.

Although comparisons of studies investigating gut microbiota composition are often challenging, five out of eight studies described above, including in vitro studies, showed an increase in fecal bifidobacteria by supplementing milk polar lipids at different concentrations. Strains of Bifidobacterium are commonly used as probiotic agents [115,116,117,118] and have been shown to have preventive and therapeutic effects in infant gut diseases [119] and in respiratory and gastrointestinal disorders in adults [120]. If supported in human studies, milk PLs may have potential as prophylactic or therapeutic agents against these diseases. It is quite interesting to note the differences in the changes in Firmicutes/Bacteroidetes ratio by the supplementation of milk PLs and milk SM. Norris et al. [10] reported a decrease in Bacteroidetes and increase in Firmicutes (hence an increased Firmicutes/Bacteroidetes ratio) by supplementing 0.25% milk SM for four weeks to HFD-fed mice. It is noteworthy that SLs can be produced in the gut by a small fraction of bacteria belonging to the Bacteroidetes phylum. A plausible justification may be that chronic exogenous SM supplementation may be triggering a feedback signaling pathway that is lethal to Bacteroidetes [63]. Another possibility may be related to greater amount of lipids getting to the colon of animals supplemented with high amounts of purified milk SM due to its noted inhibitory effects on lipid absorption. However, supplementing the same or higher amount of milk SM in the presence of other milk PLs did not change the Firmicutes/Bacteroidetes ratio [103] or decreased it [102]. The varying responses of the gut microbiota across studies may be due to differences in animal models or diets used, as well as the duration of milk polar lipid supplementation. While effects on gut microbiota composition observed at the phylum level are intriguing, more research is warranted in this area to investigate genus- and species-level compositional changes, as well as the metagenomic effects.

Recent research also suggests that choline-containing PLs can be metabolized by gut bacteria to generate trimethylamine (TMA) and, subsequently, oxidized to trimethylamine-N-oxide (TMAO) in the liver after absorption. Many observational and metabolomic studies have reported TMAO as a predictive risk factor for cardiovascular disease (CVD) [121,122] and colorectal cancer [123]. Choline, as one of the precursors for TMA, is found in many foods as free choline or as part of phosphatidylcholine, phosphocholine, or SM. Since PC is one of the most abundant polar lipids found in milk and milk products, researchers have analyzed the association between milk consumption and TMAO production. A cross-sectional study conducted in a German adult population reported a positive association between elevated plasma TMAO levels and milk consumption [124]. In contrast, another study conducted on healthy adults (KarMeN study) found no association between milk consumption and plasma TMAO [125], while intervention studies by Zheng et al. reported lower urinary TMAO levels in overweight women on high dairy diets [126] and adult men on both high milk and high cheese diets [127].

Current evidence appears insufficient to associate milk polar lipids and milk to high plasma or urinary TMAO levels. The direct effects of elevated plasma TMAO levels in promoting CVD risk is also controversial. Fish and seafoods are the rich sources of TMAO and TMA [128]. Many reports associate high fish intake to elevated plasma TMAO levels [128,129]. If Plasma TMAO can increase the risk of CVD, it should be speculated that high fish intake can increase the risk of CVD. However, epidemiological and observational studies report a protective effect of fish consumption on CVD risk [130,131,132,133]. On the other hand, fish oil has reported to ameliorate the adverse effects caused by TMAO in HFD fed mice [134]. A recent Mendelian randomization trial suggests that type 2 diabetes and kidney disease can increase circulating TMAO and evidence for the association between TMAO and CVD in observational studies may be due to reverse causality or confounding [135]. Overall, current evidence suggest choline-containing milk PLs may be used as substrates for TMA generation by gut microbiota, but the effect on disease risk is unclear. Fortunately, a number of studies have investigated the effects of milk PLs on other CVD risk factors. 

### 3.4. Cardiovascular Disease

Cardiovascular disease (CVD) remains the most prominent contributor to mortality in the United States [136]. Atherosclerosis, which is characterized by the deposition of fatty plaque in the inner walls of the arteries, is a key player in the development of CVD. Dietary modification is recommended as a primary prevention strategy for managing blood lipid levels to reduce the risk for CVD [137]. The health effects of milk PLs on serum lipid levels are summarized in Table 3 and Table 4. Genetically-obese KK-Ay mice displayed significant reductions in plasma LDL-cholesterol when fed a diet supplemented with SL-concentrated butter serum (0.35% SLs in diet by weight) or milk-derived ceramides (0.35% *w*/*w*) [138]. The feeding of milk SM (~0.25% *w*/*w* of diet) has been shown to significantly reduce serum cholesterol by ~15–25% when fed to C57BL/6 mice consuming both milk fat-enriched [10,139] and low-fat diets [63]. In humans, milk SM also shows potential to improve serum lipids. In a single-blind, randomized, controlled isocaloric parallel study, Rosqvist et al. [140] observed that an eight-week consumption of 40 g milk fat/day as whipping cream (rich in MFGM) in overweight adults resulted in lower plasma LDL-C, non-HDL-C, and apoB:apoA-I ratio compared to the same amount of milk fat as butter oil (free of MFGM). Conway et al. [141] reported that ingestion of 45 g/day of buttermilk for four weeks resulted in reductions in plasma cholesterol and TG compared to placebo, in a double-blind randomized study of healthy adults. The lower plasma LDL-C concentrations observed with buttermilk were associated with changes in plasma β-sitosterol, a marker of cholesterol absorption. In a clinical trial in postmenopausal women by Vors et al. [110], a dose of 5 g/day of milk PLs (1.3 g/day milk SM) via a butter serum concentrate lowered total and LDL-C, as well as decreased total/HDL-cholesterol and decreased apoB:apoA-I ratio, compared to a control cream cheese devoid of milk PL. Overall, it appears that the hypolipidemic effects of milk PLs observed in animal studies were also observed in several human studies, even when using lower dosages than in animal studies.

We have previously reported that supplementing purified milk SM at both 0.25% and 0.1% (*w*/*w* of diet) attenuated dyslipidemia and inflammation in HFD-fed C57BL/6J mice. Our research group [142] and others [143] have previously reported that dietary egg SM could attenuate atherosclerosis development in apoE^−/−^ mice, even in the absence of changes in serum lipids. In a recent study, we report that supplementation of 2% (*w*/*w*) milk PLs to LDLr^−/−^ mice fed a milk fat-rich diet strongly reduced atherogenic lipoprotein cholesterol, modulated gut microbiota, modestly lowered inflammatory markers, and markedly attenuated atherosclerosis development [102]. Milk polar lipids were provided by supplementing diets with butter serum, a dairy co-product rich in both PLs and SLs. Thus, due to potential beneficial effects on both serum lipids and inflammation, milk polar lipids may be important to consider when choosing foods for the prevention of CVD.

### 3.5. Non-Alcoholic Fatty Liver Disease (NAFLD)

NAFLD is the most common cause of chronic liver disease worldwide [144]. In Western societies, it affects 20%–30% of the general population and over 75% of obese individuals [145]. The “two-hit hypothesis” model of NAFLD states that the disease progresses in a stepwise manner, with a “first hit” from obesity and insulin resistance resulting in hepatic lipid accumulation, i.e., hepatic steatosis. Subsequently, a “second hit” in the form of oxidative stress and inflammation promotes liver injury and fibrosis [146]. In laboratory animals, dietary SM appears useful in preventing hepatic lipid accumulation (as extensively reviewed previously by Norris and Blesso [15]). The health effects of milk PLs on hepatic lipid metabolism are summarized in Table 3. Previous research in our laboratory has reported that HFD-fed mice supplemented with milk SM (0.25% *w*/*w*) had reduced hepatic TG after fouur weeks, compared to HFD-fed control animals [10]. In addition, our experiments investigating 10-week supplementation of a lard-based HFD (31% lard, 0.15% cholesterol by weight) with either 0.1% (*w*/*w*) milk SM or egg SM significantly attenuated the development of hepatic steatosis and adipose tissue inflammation in C57BL/6J mice [9]. Moreover, gene expression analysis revealed lower hepatic mRNA expression of stearoyl-CoA desaturase-1, indicating a reduced capacity for hepatic TG synthesis. Likewise, Cohn and colleagues have also noted that chronic supplementation of Western-type diets (21% butter fat, 0.15% cholesterol by weight) with various milk PL extracts (0.25–0.35% SM *w/w* of diet) significantly attenuated hepatic cholesterol and TG accumulation in mice. Recently, we have reported that supplementing milk fat-based diets (21% milk fat by weight) with milk PLs (at 1% and 2% *w*/*w*) resulted in significantly lower hepatic cholesterol concentrations in LDLr^−/−^ mice, although no effects were seen in hepatic TG content [102]. For human studies, there is limited data available evaluating the effects of milk PLs on NAFLD-related markers, although one study by Weiland et al. [147] found beneficial effects of 2–3 g/day of milk PLs for 7–8 weeks on serum γ-glutamyl transferase (GGT), a marker of fatty liver disease, with no changes in alanine transaminase and aspartate transaminase (markers of liver injury) in two separate clinical trials of overweight or obese men.

### 3.6. Insulin Resistance and Type 2 Diabetes

Insulin resistance occurs when the body’s cells cannot effectively import glucose in response to the release of endogenous or exogenous insulin within the bloodstream [162]. Insulin resistance can cause hyperglycemia, which makes it a risk factor for the development of type 2 diabetes mellitus (T2DM). Diabetes is a major cause of death in the U.S. and, also, contributes to significant comorbidities related to micro- and macrovascular complications, including CVD, NAFLD, kidney disease, and blindness [163]. While studies specifically examining the effects of milk polar lipids in T2DM models are lacking, the health effects of milk polar lipids on insulin resistance and glycemia are summarized in Table 5.

Nagasawa et al. [164] observed the effects of dihydrosphingosine (DHS) on activating GPR120, a receptor expressed by enteroendocrine cells that promote the secretion of the incretin GLP-1. DHS, along with phytosphingosine, was shown to strongly activate GPR120 in vitro, although sphingosine did not. Interestingly, milk SM is known to have more saturated sphingoid backbones than other SM sources [165] and, thus, would provide DHS as a hydrolytic digestive product. GLP-1 is known to have inhibitory effects on insulin resistance and T2DM; thus, more research should investigate the effects of milk polar lipids on regulating incretin production.

In contrast to in vitro effects, results from in vivo studies have been less promising. Yamauchi et al. [153] studied the effects of SM supplementation in male obese/diabetic KK-Ay mice. Mice that were supplemented with 1% (*w*/*w*) milk SM in several low-fat diets (7% by weight as lard, soybean oil, or linseed oil) for four weeks showed no significant effects in body weight, adiposity, or blood glucose compared to control groups. Correspondingly, there were no significant differences in blood glucose concentrations when measured at various time points (days 0, 7, 14, 21, and 28). Similarly, Norris et al. [9] reported that blood glucose and HOMA-IR were not significantly affected by 0.1% (*w*/*w*) milk SM supplementation in C57BL/6J mice fed an obesogenic HFD for 10 weeks, although egg SM was shown to significantly reduce fasting glucose in the same study. Weiland et al. [147] observed the effects of three different milk interventions administered to overweight and obese men. Within this report, there were two double-blind parallel-group trials that occurred involving PL-enriched milk supplementation in overweight/obese men. Trial 1 consisted of administering milk enriched with 2 g milk PL or 2 g milk fat (control) to 62 male participants over an eight-week time period. Trial 2 consisted of administering milk enriched with 3 g milk PL or 2.8 g soy PL to 57 male participants over a seven-week time period. The overall results showed a reduction in waist circumference in participants that received 2 g of milk-PL (intervention) in Trial 1 when compared to those that received the control. However, there were no differences in fasting glucose, insulin, or the insulin sensitivity index in both trials.

### 3.7. Cognitive Function and Neurodevelopment

There is a growing interest in the potential health benefits of milk PLs on neurodevelopment. The development of an infant’s brain starts at two weeks of conception and stops when the individual has reached about 20 years of age or early adulthood [166]. As galactosylceramide (cerebroside) content of myelin is important for central nervous system function [167], it is hypothesized that milk polar lipids may influence cognition when introduced into the diet at a young age. The health effects of cow’s milk polar lipids on cognitive function and neurodevelopment are summarized in Table 6.

Oshida et al. [167] studied the effects of cow’s milk SM supplementation on l-cycloserine (LCS) (an inhibitor of myelination) treatment in male Wister rat pups. Rat pups were divided into two treatment groups that were either administered LCS treatment only (control) or LCS + cow’s milk SM (0.81% *w/w* of diet) for 28 days. This study found an increase in brain weight and CNS myelin dry weight in the LCS + SM group when compared to the LCS only group. Gurnida et al. [168] examined the effects of infant formula supplemented with complex milk lipids and gangliosides on Griffith Scale values and serum ganglioside levels in infants. The treatment group consisted of infants that were given a supplemented infant formula with complex milk lipids and gangliosides (11–12 µg/mL) (derived from cow’s milk), while the control group consisted of infants that were given unsupplemented, standard infant formula. The reference group consisted of infants given breastmilk only. The infants received the treatment or control products starting at two to eight weeks of age and consumed them until six months of age. Overall, findings showed an increase in Griffith Scale values within the treatment group when compared to the control group. Correspondingly, the Griffith scale values were comparable to the values of a reference group of breastfed infants. Additionally, an increase in serum GM3, GD3, and total ganglioside levels were observed in the treatment group when compared to the control group.

### 3.8. Colorectal Cancer and Colitis

While milk polar lipids, particularly SM and other SLs, have shown promise in controlling inflammation and modulation of gut microbiota, there has also been interest in their effects on chronic diseases of the GI tract, such as colorectal cancer and inflammatory bowel disease (IBD). Colorectal cancer is the second leading cause of cancer-related deaths in the United States [169]. Meanwhile, IBD has rapidly expanded in both Western civilizations and newly industrialized nations in the 21st century [170]. In North America, over 1.5 million people suffer from IBD, which includes ulcerative colitis (UC) and Crohn’s disease (CD) [170]. These two diseases of the gut are linked, as patients with IBD have a greater risk of developing colorectal cancer [171]. The health effects of milk PLs on colorectal cancer and colitis are summarized in Table 7.

Kutchta-Noctor et al. [173] observed the effects of buttermilk, containing SM, lactosylceramide (LacCer), and ceramide, on growth inhibition of SW480 human colon cancer cells and noncancerous fetal human colon (FHC) cells. They reported that buttermilk containing SM and LacCer led to growth inhibition of SW480 cells and was selective towards cancer cells, with no effect on FHC cell growth. Early experiments reported decreased aberrant crypt foci formation in CF1 mice treated with 1,2-dimethylhydrazine (DMH) when supplemented with milk SLs in the diet [174,175,176]. In terms of the control groups, CF1 mice were fed a semi-purified diet (A1N76A) consisted of 0–0.005% SL content throughout the various studies [174,175,176]. A 66–70% decrease in aberrant colonic crypt appearance was observed by Schmelz et al. [176] when mice were supplemented with 0.1% SM by weight. Similar findings were observed by Dillehay et al. [175], who reported decreased incidence of DMH-induced colon tumors in mice fed 0.05% milk SM (*w*/*w*) when compared to the control group. Schmelz et al. [174] showed that DMH-injected CF1 mice supplemented with 0.025%–0.1% (*w*/*w*) of glucosylceramide (GluCer), LacCer, or GD3 had a measurable decrease in aberrant crypt foci formation of >40%. When compared to milk SM supplementation, GluCer, LacCer, and GD3 supplementation yielded similar decreases in aberrant crypt foci formation in DMH-injected CF1 animals. Snow et al. [177] reported a significant reduction in aberrant crypt foci when AMF-containing diets were supplemented with MFGM in Fischer-344 rats. Mazzei et al. [77] studied the effects of 0.1% (*w/w*) milk SM supplementation on azoxymethane (AOM) and dextran sulfate sodium (DSS)-induced colitis and colon tumor formation in PPARγ^+/+^ and PPARγ^−/−^ C57BL/6 mice. Compared to AOM + DSS control animals, those fed milk SM had reductions in disease activity index and colonic inflammatory lesions, with greater effects in PPARγ-expressing animals. Additional findings show decreased AOM-induced colon tumors only in PPARγ^−/−^ mice fed SM. Thus, pre-clinical studies show promising effects of milk SLs on attenuating colitis and colorectal cancer. Clinical studies are necessary to find out if these favorable effects also translate to humans.

## 4. Gaps in Scientific Literature and Future Directions

Significant research has been conducted to understand the health benefits of phytochemicals, whereas less is known about zoochemicals, such as SM found in milk polar lipids. Our research laboratory as well as others have shown in animal studies that milk polar lipids may impart health benefits through lowering blood cholesterol, inflammation, and altering gut bacteria. However, very little research has been conducted to investigate how milk polar lipids affect lipoprotein profiles, inflammation, and gut health in men and women at risk for CVD. Vors et al. [110] recently reported that postmenopausal women who consumed 5 g/day of milk polar lipids had beneficial effects on plasma lipids and increased the coprostanol/cholesterol ratio in feces. While crucial data were revealed with this important study, there are many questions that remain. For example, (1) what are the impacts of chronic intake of milk polar lipids on systemic and intestinal inflammation? Additionally, (2) how are lipoprotein particle characteristics that are known predictors of CVD risk (e.g., LDL size and HDL particle number) affected by milk polar lipids? While Vors et al. [110] did not observe changes in major bacterial phyla composition after four weeks (i.e., who is there), the increased coprostanol conversion in feces with milk polar lipid consumption suggests significant changes in gut microbiome metabolic capacity; thus, (3) are there effects of milk polar lipids on the metagenome of gut bacteria (i.e., what they are doing)? Finally, while the study by Vors et al. [110] was conducted in moderately hyperlipidemic, overweight post-menopausal women, (4) do milk polar lipid health effects differ between men and women? Further research should address these important unresolved questions.

It is quite challenging to arrive at a comparative assessment and extrapolate in vivo experiments across different mammalian models. Apart from the category of polar lipid used in different studies, some of the confounding factors could be partly due to the differences in diet, dosage, and duration of the studies across different species. A significant number of studies reviewed in this paper used SM, SLs, milk PLs, total polar lipids, or MFGM as the source of milk polar lipids. When interpreting the results of studies that used total polar lipids or milk PL, the likelihood of the synergistic effects of different polar lipids should be considered. Unlike SM studies, the effect of SLs in animal models could also be attributed to the various fractions involved. Likewise, in studies that used MFGM the effect of membrane proteins, glycoproteins, and gangliosides should be considered as potential bioactive compounds influencing the results.

## 5. Conclusions

**Figure 2:**
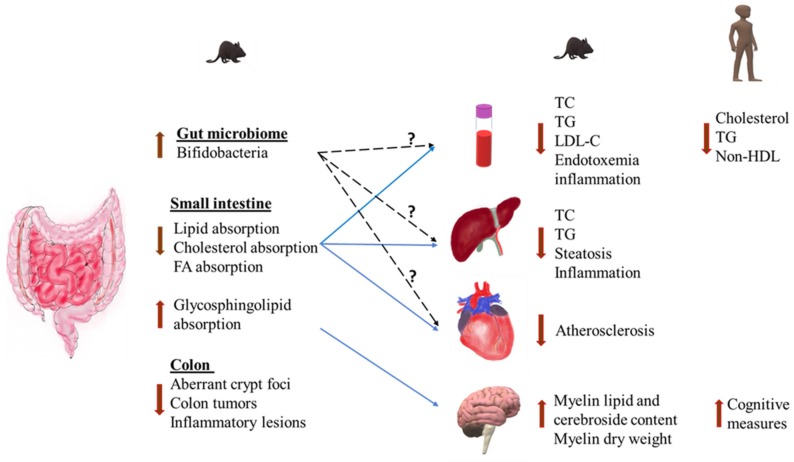
Functional properties of dietary milk polar lipids on various organs. Dietary milk polar lipids appear to have local effects in the GI tract on gut microbiome, colon health, and lipid absorption. Although the reported effects of milk polar lipids on gut microbiome are quite variable, a consistent finding in most studies is an increase in bifidobacterial population. Broken black lines indicate the hypothetical contribution of gut modulating effect of milk polar lipids on changes seen in other organs. Solid blue lines indicate the known underlying mechanisms by which milk polar lipids exert their systemic effects. Abbreviations: FA, fatty acids; TC, total cholesterol; TG, triglycerides; LDL-C, low-density lipoprotein-cholesterol; non-HDL, non-high-density lipoproteins.

In this review, we evaluated the potential health effects of milk PLs in humans by examining in vitro and in vivo studies. Milk PLs were shown to favorably influence health in relation to inflammation, CVD, NAFLD, gastrointestinal diseases, and neurodevelopment, with most effects observed in pre-clinical studies (Figure 2). As described above, this can be attributed to the much lower dosages used in human studies when compared to animal studies and more clinical studies with higher doses are needed to confirm these effects in humans. Evidence from such studies may further support the development of “designer” dairy products rich in milk PLs and SLs to enhance value and promote health. Additionally, inexpensive dairy co-products rich in PLs, such as butter serum or buttermilk, could be promoted and utilized as value-added sources of milk PLs. Overall, Milk PLs are emerging as commonly consumed dairy matrix components that may be important to consider when planning diets for the prevention of chronic disease.

## Figures and Tables

**Table 1:** Milk PL and SM content of raw milk and dairy products.

Product	PL (g/100 g DM)	PL (g/100 g Fat)	PE (% of Total PL)	PI (% of Total PL)	PS (% of Total PL)	PC (% of Total PL)	SM (% of Total PL)	Reference
Whole milk	0.2–0.3	0.7–2.3	23.2–72.2	1.4–7.5	3.4–24.5	8.0–46.4	4.0–29.5	[20,30,31,32,33,34,39,40,41,42,45]
Skim milk	0.1	10.7–11.1	26.7–38.2	5.5–8.4	8.4–9.9	19.6–35.2	16.7–21.2	[30,32]
Cream	0.2–0.4	0.3–5.6	17.7–45.6	6.8–15.4	6.7–14.8	14.6–33.7	11.9–28.6	[19,30,32,42,43,44,46]
Butter	0.3	0.2–5.31	17.7–43.3	4.3–15.8	7.0–15.3	19.9–35.6	16.6–21.8	[30,31,32,43,44,46]
Buttermilk	1.1- 2.0	4.5–35.3	17.0–44.8	2.4–17.3	8.0–18.0	17.3–46.0	12.1–21.5	[29,30,37,42,44,47,48,49]
Butter serum	11.5	46.7–48.4	26.7–31.4	9.0–11.2	6.9–10.1	24.9–27.2	23.8–28.9	[30,32,47]
Cheese whey	0.3–1.8	5.3–23.7	27.4–41.1	2.8–3.7	3.9–9.3	19.0–32.2	9.9–16.4	[29,30,48,50]
Yogurt (skimmed)	0.2	5.5	31.1	6.3	7.9	19.9	24.9	[30]
Ricotta cheese	1.16	2.7	45.4	4.4	5.8	15.8	14.2	[30]
Mozzarella cheese	0.28	0.5	42.5	5.7	5.6	19.4	14.6	
Cheddar cheese	0.25	0.5	38.0	7.7	8.5	20.3	16.3	

Abbreviations: DM, dry matter; PC, phosphatidylcholine; PE, phosphatidylethanolamine; PI, phosphatidylinositol; PL, phospholipid; PS, phosphatidylserine; and SM, sphingomyelin.

**Table 2:** Animal and human studies examining the effects of milk polar lipids on gut microbiota.

Authors	Model	Control	Treatment	Duration	Results	Reference
Reis et al. (2013)	C57BL/6J mice	HFD (*n* = 13)	HFD followed by supplementation of total polar lipids (TPL), phospholipids (PL), or sphingolipids (SPL) through HFD (*n* = 13)	5 weeks on HFD followed by 5 weeks on TPL/PL/SM	Little effect of the polar lipid dietary supplementation on the composition of cecal microbiota was observed (*p* > 0.05).	[107]
Nejrup et al. (2015)	Fecal samples from nine healthy infants (aged 2–5 months)		Medium chained and long chain NEFA with and without 10 mol% sphingosine	24 h in vitro	LC-NEFA with sphingosine: increased bifidobacteria	[99]
Zhou et al. (2018)	21-d-old Sprague–Dawley rats	0 µM Ethanolamine in drinking water	250, 500 and 1000 μM Ethanolamine from milk in drinking water for 2 weeks	2 weeks	Increased: Bacteroidetes (500 and 1000 μM) Decreased: Proteobacteria, Elusimicrobia and Tenericutes (500 and 1000 μM) Spirochetes (500 μM)	[109]
Norris et al. (2016)	Male C57BL/6 mice	HFD (21% added milk fat by weight) (*n* = 3)	0.25% (*w/w*) milk SM in HFD (*n* = 10)	4 weeks	Increased: Firmicutes, bifidobacteria, Actinobacteria and Gram-positives Decreased: Bacteroidetes, Tenericutes and Gram-negatives	[9]
Norris et al. (2017)	Male C57BL/6 mice	HFD (31% lard; 0.15% cholesterol by weight) (*n* = 14)	0.1% (*w/w*) milk SM in HFD. (*n* = 14)	10 weeks	Increased Acetatifactor	[11]
Bhinder et al. (2017)	5 to 15 days old Rats (Used pup in a cup model)	Fed with mothers’ milk (MM)	Formula with MFGM comprising part of the fat component or Formula with fat derived entirely from vegetable source	15 days	MFGM formula: microbial richness and evenness similar to MM. Similar abundances of Firmicutes and Proteobacteria compared to MM MFGM formula: Increased Lactobacilli, Enterococcus, Clostridiales, Streptococcus, and Morganella vs. vegetable fat formula.	[108]
Li et al. (2018)	5 weeks old C57BL/6J mice	Chow diet (*n* = 10)	HFD (*n* = 10) or HFD + MFGM (Lacprodan^®^ MFGM-10) at 200 mg/kg BW (*n* = 10)		MFGM diet increased the relative abundance of Porphyromonadaceae, S24–7, norank_f_Bacteroidates_S24- 7_group, unclassified_f_Lachnospiraceae, and Odoribacter compared with the HFD group Increased ACE index compared with HFD. MFGM supplementation recovered 13 key genera found enriched in control group Simpson’s index showed no difference among three group	[84]
Milard et al. (2019)	Male C57BL/6J mice	HFD (21% *w*/*w* palm oil in chow)	8 weeks on HFD with 1.1% (*w*/*w*) milk PL or 1.6% (*w*/*w*) of milk PL	8 weeks	Increased: *Bifidobacterium*, in particular *Bifidobacterium animalis* in 1.1% of milk PL group Decreased: Lactobacillus in 1.6% of milk PL group Positive correlation between *Bifidobacterium animalis* and *Akkermansia* *muciniphila*	[103]
Vors et al. (2019)	Double-blind, parallel clinical trial in 58 Overweight postmenopausal women	No milk PL via butter serum (*n* = 19)	3 mg (*n* = 19) or 5 mg (*n* = 20) of milk PL via butter serum	4 weeks	No change in major phylogenetic groups and bacterial species of gut microbiota Increased fecal coprostanol/cholesterol ratio	[110]
Millar et al. (2020)	LDLr^−/−^ mice	HFD (45%) for (*n* = 15)	HFD (45%) with 1% or 2% milk PL (MPL) (*n* = 15)	14 weeks	2% MPL: Increased Actinobacteria, Bacteroidetes, *Bifidobacterium*, Bacteriodales_unclassified. Reduced Firmicutes/Bacteroidetes ratio 1% MPL: Increased Shannon diversity	[102]

Abbreviations: ACE, abundance based coverage estimator; BW, body weight; HFD, high-fat diet; LC-NEFA, long-chain non-esterified fatty acids; LDLr, low-density lipoprotein receptor; MM, mother’s milk; MFGM, milk fat globular membrane; NEFA, non-esterified fatty acids; PL, phospholipid; SM, sphingomyelin, SPL, sphingolipids; and TPL, total polar lipids.

**Table 3:** Animal studies examining the effect of milk polar lipids on serum and hepatic lipids.

Authors	Animal Model	Control	Treatment	Duration	Results	Reference
Nyberg et al. (2000)	Male Sprague-Dawley rats (*n* = 5–8)	Cholesterol mixed in soybean oil (without PL)	2.6:1, 1:1 or 0.5:1 molar ratio of cholesterol:SM	3 days	Decreased intestinal cholesterol absorption (lowest in cholesterol:SM ratio 1:1)	[148]
Eckhardt et al. (2002)	Male C57BL/6 mice (*n* = 6)	Chow	Chow diet enriched in PL (containing 0.1%, 0.5% or 5% of milk SM by weight)	4 days	Decreased intestinal cholesterol absorption	[64]
Wat et al. (2009)	Male C57BL/6 mice (*n* = 10)	LFD or HFD without milk PL	LFD or Western-type diet with 1.2% (*w*/*w*) PL from phospholipid-rich dairy milk extract (PLRDME)	8 weeks	Serum lipids: PLRDME with western-type diet group: Decreased TG (−20%), phospholipids (−21%) and HDL-C (−19%) PLRDME with LFD group: No change in TC, TG, phospholipids and HDL-C Hepatic Lipids: PLRDME with western-type diet group: Decreased total lipid (−33%), TG (−44%), TC (−48%) and phospholipids (−16%) PLRDME with LFD group: No change in total lipid, TG, TC and phospholipids	[139]
Kamili et al. (2010)	Male C57BL/6 mice (*n* = 10)	Western-type diet without milk PL	Western-type diet (21% AMF; 0.15% cholesterol by weight) with 1.2% (*w*/*w*) PL from PLRDME or milk phospholipid concentrate (PC-700)	3, 5 or 8 weeks	Plasma lipids: PLRDME after 8 weeks: Decreased plasma TC (−23%) Hepatic lipids: PLRDME after 5 weeks: Decreased total lipid (−41%), TG (−47%) and TC (−39%) PLRDME after 8 weeks: Decreased total lipid (−18%) and TG (−28%) PC−700 after 5 weeks: Decreased total lipid (−45%), TG (−63%) and TC (−57%)	[149]
Watanabe et al. (2011)	Female KK-A^y^ mice (*n* = 7)	AIN-93G diet	AIN-93G diet with 1.7% (*w*/*w*) of lipid-concentrated butter serum (LC-BS) or 0.5% (*w*/*w*) of ceramide-rich fraction (Cer-fr) or 0.5% (*w*/*w*) of SM-rich fraction (SM-fr)	4 weeks	Plasma lipids: SM-fr: no change LC-BS: Decreased TC (−18%) and LDL-C (−45%) Cer-fr: Change only in TC (−25%) Hepatic lipids: SM-fr: No change LC-BS: Decreased TG (−27%) Cer-fr: Decreased TG (−38%) and TC (−47%)	[138]
Zhou et al. (2012)	Fischer-344 rats (*n* = 3–4)	AIN-76A diet with corn oil or AMF (0.5% *w*/*w*)	2.5% (*w*/*w*) MFGM, 2.5% (*w*/*w*) AMF in AIN-76A diets	12 weeks	Decreased esterified cholesterol and increased TG in liver	[150]
Reis et al. (2013)	Male C57BL/6 (*n* = 13)	HFD	HFD (~20% lard by weight) with 1.7% (*w*/*w*) total polar lipids extracts or 1.4% (*w*/*w*) phospholipids-rich extract or 0.4% (*w*/*w*) SM-rich extract	5 weeks	Decreased FA synthesis in liver by total PL extract and PL-rich extract Decreased 16:1n-7/16:0 in liver by SM-rich extract	[107]
Lecomte et al. (2015)	Female Swiss mice (*n* = 7)	Emulsion with soybean PL (gavaged)	Emulsion with 5.7 mg milk PL (gavaged)	1, 2 or 4 h	After 1 h: Increased plasma NEFA and a trend to increase TG After 4 h: Decreased plasma TG and NEFA associated with a decreased duodenal gene expression of APOB 48 and Sar1b	[151]
Norris et al. (2016a)	Male C57BL/6 mice (*n* = 10)	HFD (21% AMF by weight)	0.25% (*w*/*w*) milk SM in HFD (21% AMF by weight)	4 weeks	Decreased serum TC and hepatic TG No change in serum TG and hepatic TC	[10]
Norris et al. (2017)	Male C57BL/6 mice (*n* = 14)	HFD (31% lard; 0.15% cholesterol by weight)	0.1% (*w*/*w*) milk SM in HFD	10 weeks	No change in serum lipids Decreased hepatic TC (−23%) and TG (−30%)	[9]
Lecomte et al. (2016)	Male C57BL/6J mice (*n* = 10–12)	HFD (17% *w/w* palm oil) + soybean PL	1.2 % (*w*/*w*) milk PL or SPL in HFD (17% *w*/*w* palm oil)	8 weeks	No change in plasma and hepatic lipids Increased fecal VLCFA such as C22:0, C24:0 and C22:4(*n*-6)	[152]
Yamauchi et al. (2016)	Obese/diabetic KK-A^y^ (*n* = 7) and male C57BL/6 mice (*n* = 6)	HFD (lard, soybean, linseed or fish)	1% (*w*/*w*) milk SM in HFD (lard, soybean, linseed or fish)	4 weeks	No effect on wild type mice In KK-A^y^ mice: Soybean + SM: decreased serum LDL-C and non-HDL-C. Increased hepatic total lipids, cholesterol, bile acid. Linseed + SM: Decreased serum LDL-C. Decreased hepatic total FA and increased fecal total lipid and cholesterol. Lard + SM: Increased fecal total lipids, cholesterol, and decreased hepatic total FA.	[153]
Zhou et al. (2019)	Male ob/ob mice (*n* = 11–18)	Moderately high-fat AIN-93G diet (34% kcal as fat) without milk PL or gangliosides	(0.2% (*w*/*w*) milk gangliosides (GG) or 1% (*w*/*w*) milk PL (PL) in moderately high-fat AIN-93G diet (34% kcal as fat)	2 weeks	No change in plasma and hepatic lipids by milk GG. PL increased plasma NEFA, PL, SM and DAG and decreased hepatic CE.	[154]
Millar et al. (2020)	LDLr^−/−^ mice	HFD (45%) for (*n* = 15)	HFD (45%) with 1% or 2% milk PL (MPL) (*n* = 15)	14 weeks	2% MPL: Decreased serum cholesterol (−51%), with dose-dependent reduction in VLDL-C and LDL-C. Decreased hepatic TC (−55%) 1% MPL: Decreased hepatic TC (−53%)	[102]

Abbreviations: AMF, anhydrous milk fat; ApoB, apolipoprotein B; Cer-fr, ceramide-rich fraction; CE, cholesteryl ester; DAG, diacylglycerol; FA, fatty acid; GG, gangliosides; HDL-C, high-density lipoprotein cholesterol; HFD, high-fat diet; LC-BS, lipid-concentrated butter serum; LDL-C, low-density lipoprotein cholesterol; LFD, low-fat diet; MFGM, milk fat globular membrane; NEFA, non-esterified fatty acids; PL, phospholipids; PLRDME, phospholipid-rich dairy milk extract; Sar1B, secretion-associated: SM, sphingomyelin; SM-fr, sphingomyelin-rich fraction; SPL, soybean polar lipids; TC, total cholesterol; TG, triglyceride; and VLCFA, very long-chain fatty acids.

**Table 4:** Human clinical trials examining the effects of milk polar lipids on serum lipids.

Authors	Population and Study Design	Control	Treatment	Duration	Results	Reference
Ohlsson et al. (2009)	Parallel group study with 33 healthy men and 15 healthy women	119 mg of total SL (isocaloric)	2 drinks/day totaling 975 mg SL containing 700 mg SM, 180 mg GC and 95 mg GS	4 weeks	No change in plasma lipids. Trend for decreasing LDL-C (only in women)	[155]
Ohlsson et al. (2010)	Human ileostomy contents from 6 men and 6 women		1. Milk SM (250 mg) mixed in skimmed milk 2. Milk SM (50,100 or 200 mg) mixed in milk-like oat drink	Collected after 8 h	Increased the out-put of VLCFA specific of milk SM (22:0, 23:0, 24:0)	[156]
Ohlsson et al. (2010)	Crossover study in 18 healthy adult males	High-fat (40 g) standard breakfast together with a milk-like formulation lacking polar milk lipids	High-fat (40 g) standard breakfast together with a milk-like formulation containing 975 mg of milk SL	1 to 7 h	No change in plasma lipids after 1 h Trend for decreasing cholesterol in large TG- rich lipoproteins.	[157]
Keller et al. (2013)	Parallel study in 14 healthy women	Baseline	2 supplementation cycle–3 g milk PL/day followed by 6 g milk PL/day	10 days each	3 g milk PL: Decreased plasma TC, HDL-C After 6 g milk PL supplementation: Increased plasma TC and LDL-C	[158]
Conway et al. (2013)	Double-blinded crossover study in 34 healthy adults	45 g/day of a macro/micronutrient matched placebo	45 g buttermilk powder/day	4 weeks	Decreased serum cholesterol (−3.1%), TG (−10.7%) and trend for decreasing LDL-C (*p* = 0.057) Decreased LDL-C (−5.6%) in participants with highest (top 50%) baseline LDL-C	[141]
Baumgartner et al. (2013)	Single-blind parallel study in 97 healthy adults	One or two eggs a week (*n* = 20)	1. One egg/day (*n* = 57) 2. 100 mL/day of buttermilk drink containing one egg yolk (*n* = 20)	12 weeks	No difference in serum lipids, liver inflammatory markers, Apo-A1, Apo-B100, campesterol, or lathosterol between the two treatment groups	[159]
Rosqvist et al. (2015)	Single-blind, parallel study in 57 overweight adults	Butter oil (1.3 mg total PL), matched for calories, macronutrients, and calcium	40 g milkfat/day as whipping cream (198 mg total PL)	8 weeks	Decreased plasma cholesterol, LDL-C, non-HDL-C, and apoB:apoA1 ratio	[140]
Severins et al. (2015)	Single-blind, parallel study in 92 mildly hypercholesterolemic adults	80 mL of skim-milk powder (*n* = 25)	1. 80 mL skim-milk with lutein enriched egg yolk (28 g from 1.5 eggs providing 323 mg cholesterol) 2. Buttermilk (72 mg PL) 3. Buttermilk with lutein enriched egg yolk (28 g from 1.5 eggs providing 323 mg cholesterol)	12 weeks	Buttermilk addition could not change the increased serum lipids levels due to of egg yolk Buttermilk group showed a trend for decreasing TC (*p* = 0.077), but not for LDL-C	[160]
Weiland et al. (2016)	Double-blind parallel-group intervention trials in overweight or obese males.	Milk enriched with 2 g milk fat (*n* = 31)	Milk enriched with 2 g milk PL (*n* = 31)	8 weeks	Decreased GGT and waist circumference No change in plasma lipids (total, HDL- and LDL-cholesterol, total cholesterol:HDL-cholesterol ratio, TAG, PL), ALT, AST, apoB, apoA1, glucose, insulin, insulin sensitivity index, C-reactive protein, IL-6, soluble intracellular adhesion molecule and total homocysteine (tHcy).	[147]
		Milk enriched with 2.8 g soy PL (*n* = 57)	Milk enriched with 3 g milk PL (*n* = 57)	7 weeks	Decreased only GGT No change in plasma lipids (TC, HDL-C, LDL-C, TG, phospholipids, TC:HDL-C ratio), apoA1, apoB, glucose, insulin, HOMA-IR, hs-CRP, IL-6, sICAM, ALT, AST	
Grip et al. (2018)	Double blinded study in formula fed infants.	Breast fed infants (*n* = 80)	Formula without MFGM (*n* = 160)	4, 6 and 12 months	Decreased plasma PC and SM	[161]
Vors et al. (2019)	Double blinded parallel study in 58 postmenopausal women	No milk PL via butter serum (*n* = 19)	3 g (*n* = 19) or 5 g (*n* = 20) of milk PL via butter serum	4 weeks	Decreased fasting total cholesterol, LDL-C, TC/HDL-C ratio, ApoB/ApoA1 ratio, post-prandial total cholesterol, chylomicron lipids.	[110]
	Double blind cross-over study in 4 ileostomized subjects	No milk PL via butter serum (*n* = 19) with ^2^H-cholesterol tracer	3 g (*n* = 19) or 5 g (*n* = 20) of milk PL via butter serum with ^2^H-cholesterol tracer	Acute post-prandial	Decreased ^2^H-cholesterol tracer in plasma and chylomicrons. Increased ileal output of total cholesterol and of milk SM	

Abbreviations: ALT, alanine transaminase; ApoA-I, apolipoprotein A-I; ApoB, apolipoprotein B; AST, aspartate transaminase; GC, glucosylceramide; GGT, gamma-glutamyl transferase; GS, gangliosides; HDL-C, high-density lipoprotein cholesterol; HOMA-IR, homeostatic model assessment of insulin resistance; hs-CRP, high-sensitivity C-reactive protein; IL-6, interleukin -6; LDL-C, low-density lipoprotein cholesterol; MFGM, milk fat globule membrane; PC, phosphatidylcholine; PL, phospholipids; sICAM, soluble intercellular adhesion molecule; SL, sphingolipids; SM, sphingomyelin; TAG, triacylglycerol; TC, total cholesterol; TG, triglyceride; and VLCFA, very long chain fatty acid.

**Table 5:** Studies examining effects of milk polar lipids on insulin resistance and type 2 diabetes.

Author	Model	Control	Treatment	Duration	Results	Reference
Nagasawa et al. (2018)	293 T cells		Dihydrosphingosine or phytosphingosine or sphingosine	24 h	Significant upregulation of GPR120 (a receptor for long chain fatty acids) by dihydrosphingosine and phytosphingosine. Sphingosine - no effect.	[164]
Yamauchi et al. (2016)	obese/diabetic KK-*A^y^* mice (*n* = 7)	Lard or soybean oil or linseed oil	Lard + 1% SM or soybean oil +1% SM or linseed oil + 1% SM	4 weeks	No difference in blood glucose level	[153]
	wild-type C57BL/6J mice (*n* = 7)	Linseed oil or fish oil or lard + soybean oil	Linseed oil + 1% SM or fish oil + 1% SM or lard + soybean oil + 1% SM	4 weeks	No difference in blood glucose level	
Weiland et al. (2016)	Double-blind parallel-group intervention trials in overweight or obese males.	Milk enriched with 2 g milk fat (*n* = 31)	Milk enriched with 2 g milk PL (*n* = 31)	8 weeks	No difference in blood glucose, insulin and HOMA-IR between groups	[147]
		Milk enriched with 2.8 g soy PL (*n* = 57)	Milk enriched with 3 g milk PL (*n* = 57)	7 weeks	No difference in blood glucose, insulin and HOMA-IR between groups	
Norris et al. (2017)	Male C57BL/6 mice	HFD (31% lard; 0.15% cholesterol by weight) (*n* = 14)	0.1% (*w*/*w*) milk SM (*n* = 14) in HFD	10 weeks	No difference in fasting serum insulin, glucose concentrations and HOMA-IR between groups	[9]

Abbreviations: HFD, high-fat diet; HOMA-IR, homeostatic model assessment of insulin resistance; PL, phospholipids; and SM, sphingomyelin.

**Table 6:** Studies examining effects of milk polar lipids on cognitive/brain development.

Authors	Model/Population and Study Design	Control	Treatment	Duration	Results	Reference
Oshida et al. (2003)	Male Wister rat pups (*n* = 30)	No l-Cycloserine (LCS) treatment or dietary SM (non-LCS)	Daily s/c injection of 100 mg/kg of LCS from 8 days old + diet without (LCS group) or with 810 mg/100 g of SM (SM-LCS group) from 17 days old	Until 28 days old	Significantly high myelin dry weight, myelin total lipid content, and cerebroside content in the SM-LCS group than in the LCS group. Axon diameter, nerve fiber diameter, myelin thickness, and g value of optic nerve were similar in SM-LCS and non-LCS groups.	[167]
Tanaka et al. (2013)	Randomized, double-blind controlled trial in 28 premature infants with birth weight less than 1500 g	Milk (13 g SM/100 g PL) (*n* = 14)	Sphingomyelin fortified milk (20 g SM/100 g PL) (*n* = 14)	18 months	Significantly better Behavior Rating Scale of the BSID-II, Fagan test scores, latency of VEP, and sustained attention test scores	[172]
Gurnida et al. (2012)	Double-blind, parallel study in infants 2 to 8 weeks of age	Standard infant formula (0.22% milk PL and 0.006% gangliosides) (*n* = 30)	Complex lipid-supplemented formula (0.235% milk PL and 0.009% gangliosides) (*n* = 29)	From 2–8 weeks of age to until 24 weeks of age	Increased Hand and Eye coordination IQ score (*p* < 0.006), Performance IQ score (*p* < 0.001) and General IQ score (*p* = 0.041).	[168]

Abbreviations: BSID, Bayley Scales of Infant Development; LCS, 1-Cycloserine; PL, phospholipids; and SM, sphingomyelin.

**Table 7:** Studies examining effects of milk polar lipids on colon cancer and colitis.

Authors	Model	Control	Treatment	Duration	Results	Reference
Kutchta-Noctor et al. (2016)	SW480 colon cancer cells and FHC cells (normal human colon cells)	Controls contained only media. Sodium butyrate (5 mM), a potent apoptotic fatty acid, served as a positive control.	Buttermilk between 0 and 0.94 mg/mL of media	3 days at 37 ℃ in CO_2_ incubator	Inhibited growth of SW480 colon cancer cells in dose-dependent manner with selective antiproliferative activity toward cancer cells Downregulated growth signaling pathways mediated by Akt, ERK1/2, and c-myc.	[173]
Schmelz et al. (2000)	5 weeks old female CF1 mice	i/p injection of 1,2-DMH (DMH)@ 30 mg/kg body weight for 6 weeks + sphingolipid free AIN 76A diet	i/p injection of 1,2-dimethylhydrazine (DMH) at 30 mg/kg body weight for 6 weeks + AIN 76A diet with 0.025 or 0.1 g/100 g of milk GluCer, LacCer or ganglioside GD3 after 1 week	4 weeks	Glycosphingolipid groups: >40% reduction (*p* < 0.001) in appearance of aberrant crypt foci, reduced proliferation (up to 80%; *p* < 0.001) in colonic crypts.	[174]
Dillehay et al. (1994)	CF1 mice	Injection of 1,2-DMH + diet without SM	Injection of DMH + diets with 0.025 to 0.1 g/100 g of SM for 28 weeks followed by diet without SM	52 weeks	SM fed groups: 20% incidence of colon tumors (vs 47% in controls)	[175]
Schmelz et al. (1996)	5 weeks old female CF1 mice	i/p injection of 0.5 mL/kg of DMH once weekly for 6 weeks followed by diet without SM	i/p injection of 0.5 mL/kg of DMH once weekly for 6 weeks followed by diet supplemented with 0 to 0.1% (*w*/*w*) buttermilk or powdered milk SM	34 weeks	0.1% SM: Reduced appearance of aberrant colonic crypt foci (*p* < 0.001) and significantly fewer aberrant crypts per colonic focus	[176]
Snow et al. (2010)	Fischer-344 rats	i/p injection of 1,2-DMH (25 mg/kg BW) once weekly for 2 weeks followed by AIN-76A diet corn oil	i/p injection of 1,2-dimethylhydrazine (25 mg/kg BW) once weekly for 2 weeks followed by AIN-76A diet with AMF or with 50% MFGM, 50% AMF	9 weeks	MFGM group had significantly fewer aberrant crypt foci	[177]
Mazzei et al. (2011)	PPARγ^+/+^ and PPARγ^−/−^ mice	Semi-purified sphingolipid-free AIN76A diet for 7 weeks followed by single injection of azoxymethane (10 mg/kg BW).	0.1% SM (*w*/*w*) supplemented diet for 7 weeks followed by single injection of azoxymethane (10 mg/kg BW).	9 weeks	SM group of both genotypes: Decreased disease activity and colonic inflammatory lesions (more efficiently in PPARγ^+/+^ mice).	[77]

Abbreviations: AMF, anhydrous milk fat; BW, bodyweight; DMH, dimethylhydrazine; GluCer, glucosylceramide; LacCer, lactosylceramide; and SM, sphingomyelin.
